# Artificial Intelligence Perceptions and Readiness Among Clinical Nurses: A Latent Profile Analysis With Implications for Nursing Management

**DOI:** 10.1155/jonm/8080856

**Published:** 2026-09-28

**Authors:** Bei Yang, Jiacheng Hu, Qin Zeng

**Affiliations:** ^1^ Department of Pediatric Gastroenterology Nursing, West China Second University Hospital, Sichuan University, Chengdu Sichuan, 610041, China, scu.edu.cn; ^2^ Key Laboratory of Birth Defects and Related Diseases of Women and Children (Sichuan University), Ministry of Education, Chengdu Sichuan, 610041, China, scu.edu.cn; ^3^ West China School of Nursing, Sichuan University, Chengdu Sichuan, 610041, China, scu.edu.cn

**Keywords:** artificial intelligence, artificial intelligence anxiety, artificial intelligence literacy, latent profile analysis, nurses, nursing education

## Abstract

**Aims:**

This study aimed to identify latent profiles of artificial intelligence (AI) perceptions and readiness among clinical nurses based on their AI‐related attitudes, literacy, self‐efficacy, and anxiety, and to explore the influencing factors associated with each profile.

**Materials and Methods:**

Nurses were recruited via convenience sampling from public hospitals in China. A total of 1018 valid questionnaires were included for final analysis. Latent profile analysis was performed to classify subgroups. Multivariate logistic regression was adopted to explore the influencing factors of different profiles.

**Results:**

Three profiles were identified: the Low AI Perceptions and Readiness Profile (66.8%), characterized by the lowest AI attitudes, literacy, and self‐efficacy with moderate anxiety; the Ambivalent AI Perceptions and Readiness Profile (20.8%), exhibiting moderate attitudes and literacy but the highest self‐efficacy and anxiety; and the High AI Perceptions and Readiness Profile (12.4%), demonstrating the highest attitudes, literacy, and self‐efficacy with the lowest anxiety. Significant differences across profiles were found in education level, work experience, preceptor role, AI training experience, region, hospital level, and teaching hospital status.

**Conclusions:**

Clinical nurses exhibit substantial heterogeneity in AI perceptions and readiness patterns. Most are in the Low AI Perceptions and Readiness group due to insufficient training and limited resources. The Ambivalent AI Perceptions and Readiness group consists of nurses with mixed AI perceptions and high anxiety levels who require targeted support, while the High AI Perceptions and Readiness group can serve as peer mentors. These findings highlight the necessity of profile‐based training to enhance AI integration in nursing education and practice.

**Implications for Nursing Management:**

The three profiles identified in this study provide nurse administrators with an empirically based reference for differentiating educational approaches across learner groups. This classification can assist in designing targeted content, structuring peer support, and planning curricula to facilitate AI integration in nursing practice.

## 1. Background

As the healthcare industry continues to undergo digital transformation, artificial intelligence (AI) technology has gradually permeated various aspects of nursing education, clinical care, and nursing management due to its powerful capabilities in data processing, intelligent simulation, and precise analysis [1–3]. The application of AI in the nursing field not only overcomes the limitations of traditional nursing training—such as time and space constraints, content uniformity, and insufficient personalization [2, 4–6], but also provides new possibilities for further improving training quality [2]. Intelligent training platforms, virtual simulation nursing systems, and personalized learning modules can tailor training content according to individual nurses’ learning paces and skill gaps, enabling a deep integration of theoretical knowledge with practical clinical operations [7–9]. This effectively enhances nurses′ engagement in training and their efficiency in absorbing knowledge.

Simultaneously, AI technology is increasingly being integrated into clinical nursing practices, playing a critical role in areas such as patient condition monitoring [10], decision support for nursing interventions [11], health education [12], and medical record management [13]. The application of this technology not only improves the accuracy and efficiency of nursing work but also compels nurses to enhance their adaptability to AI tools and incorporate these technologies into their daily practice routines [14]. Therefore, promoting nurses’ adaptation to AI technology and standardizing their use of AI have become core goals of modern nursing training. These efforts are necessary to align with the digital transformation of healthcare and ensure the delivery of high‐quality nursing services.

Despite the widely recognized significance of AI in nursing, nurses hold highly divergent internal perceptions of AI, which may further shape their subsequent AI usage behaviors in clinical settings [15]. These differences are not merely surface‐level phenomena but are influenced by multiple factors, including intrinsic psychological cognition, competency levels, and emotional states [15, 16]. Existing studies indicate that nurses’ attitudes toward AI, AI‐related competencies, self‐efficacy in using AI technology, and AI application anxiety are core psychological dimensions reflecting nurses’ overall AI perceptions and readiness, and there are obvious individual differences across nursing populations [15, 17]. It can be inferred that some nurses may hold positive attitudes toward AI, possess adequate AI‐related competencies, and show high readiness to embrace intelligent technologies driven by strong self‐efficacy. Conversely, other nurses may hold conservative or passive attitudes toward AI, possibly due to inadequate AI skills, low self‐confidence, or anxiety related to operational difficulties. These internal differences within the nursing group make it difficult for uniform nursing training and AI promotion strategies to meet the diverse needs of all nurses, thereby hindering the deep integration of AI into nursing practice.

In light of this, precisely identifying the variations in AI perceptions and readiness among nurses and analyzing the core characteristics of these differentiated groups has become a critical prerequisite for optimizing nursing training programs and promoting the precise application of AI in nursing. Latent profile analysis (LPA), as a statistical method based on individual‐level data, can scientifically classify research subjects according to observed variables and accurately identify heterogeneous features within a population [18]. Compared with traditional grouping methods, LPA better aligns with the actual needs of individual difference research and effectively uncovers underlying patterns in the data. This study focuses on four core dimensions: nurses’ attitudes toward AI, AI competency, AI usage self‐efficacy, and AI‐related anxiety, and uses LPA to classify nurses into subgroups. It examines subgroup differences in nurses’ AI perceptions and readiness. We further explore demographic and occupational influencing factors associated with each latent profile. The aim is to clarify the heterogeneous characteristics of AI perceptions and readiness among nurses and provide empirical evidence for developing differentiated and targeted AI nursing training programs and optimizing AI promotion strategies in nursing. This effort seeks to improve nurses’ psychological acceptance and readiness for AI and facilitate the standardized and efficient implementation of AI technology in the nursing field.

## 2. Materials and Methods

### 2.1. Design and Participants

The present study employed a multicenter cross‐sectional survey design. Data were collected via convenience sampling among nurses employed at public hospitals in China from May to July 2025. Eligible participants were registered nurses who provided informed consent to voluntarily participate in the study. Nurses were excluded if they were (1) enrolled in standardized training, departmental rotations, advanced studies, or on probation; (2) retired and rehired; (3) absent from work for more than 6 months in the past year (e.g., on sick or maternity leave); (4) previously involved in similar surveys; or (5) completed the questionnaire in less than 3 min or more than 30 min.

### 2.2. Samples

The required sample size was initially estimated using the standard formula for cross‐sectional studies [19], which is expressed as follows:

(1) n=Z12‐α/δ2×p×1−p.

In the formula, *Z*_1−*α*/2_ stands for the critical value of the standard normal distribution. When the significance level *α* = 0.05, the corresponding critical value is *Z*_1−*α*/2_ = 1.96. The parameter *p* denotes the expected proportion; to maximize the sample size and ensure sample representativeness, *p* was set to 0.5. The parameter *δ* refers to the margin of error, which was defined as 0.05 in this study.

Based on the above parameters, the theoretical minimum sample size was calculated to be 385 participants. Considering potential factors such as a nonresponse rate and invalid questionnaires (e.g., incomplete responses, contradictory answers), the sample size was increased by 20% to improve the validity and reliability of the findings. Finally, the target sample size was determined to be 462 cases.

This formula was chosen to ensure precision for descriptive epidemiological estimates. For LPA, current recommendations based on Monte Carlo simulations suggest that a total sample of 300–500 participants is generally sufficient for stable model convergence and accurate class assignment when the smallest class proportion is above 10% [20, 21]. Thus, our preset recruitment target of 462 meets the empirical criteria for LPA, and we obtained a final analytical sample of 1018 valid questionnaires.

### 2.3. Presurvey

To ensure the cross‐cultural applicability of the adopted AI‐related measurement scales among Chinese nurses, this study followed a systematic validation process. After the expert panel evaluated the content validity of the Chinese‐language versions, a convenience sampling method was used to select 50 nurses for a pretest, focusing on the semantic clarity, accuracy of expression, and completion time of the questionnaire items. Based on the pretest results, language refinements and structural adjustments were made to the scales.

### 2.4. Data Collection

Data collection was coordinated by the Chinese Nursing Association. The director of the nursing department at the researchers’ affiliated hospital distributed questionnaires to national committee members of the association, who then forwarded the questionnaires to directors of nursing departments and head nurses in public hospitals; these questionnaires were ultimately distributed to registered nurses for completion. The questionnaires were administered electronically via the Wenjuanxing online platform. This study was approved by the Medical Research Ethics Committee of the researchers’ affiliated institution [Ethics approval number: Medical Research 2025 Ethics Approval No. (050)].

### 2.5. Measures

#### 2.5.1. Demographic Characteristics

The demographic data collected from participants in this study included the following items: gender, age group, ethnicity, marital status, education level, professional title, years of work experience, department, job content, whether they serve as a preceptor, AI training experience, region of affiliation, hospital type, hospital level, and whether the hospital is a teaching hospital.

#### 2.5.2. General Attitudes Toward Artificial Intelligence Scale

The General Attitudes toward Artificial Intelligence Scale [22] is a validated instrument used to assess positive and negative attitudes toward AI among individuals. This scale has also been widely adopted in nursing research to evaluate nurses’ attitudes toward AI [23]. The scale consists of two subscales: Positive Attitudes and Negative Attitudes, with a total of 20 items. All items are rated on a 5‐point Likert‐type scale. Items on the Positive Attitude subscale are scored directly (1 = *strongly disagree*, 5 = *strongly agree*), whereas items on the Negative Attitude subscale are reverse‐scored (5 = *strongly disagree*, 1 = *strongly agree*). Total scores range from 20 to 100, with higher scores reflecting more positive attitudes toward AI. In the present study, the Cronbach’s *α* coefficient for this scale was 0.890. Additionally, the scale’s Kaiser–Meyer–Olkin (KMO) value was 0.947, and Bartlett’s test of sphericity showed a significance level of < 0.001, indicating excellent internal consistency and structural validity for the adapted Chinese version, making it suitable for this study.

#### 2.5.3. Artificial Intelligence Literacy Scale

The Artificial Intelligence Literacy Scale [24] is regarded as an ideal instrument for assessing AI literacy, owing to its proposed four‐dimensional structural model encompassing awareness, usage, evaluation, and ethical capability. This scale is designed to quantify the core competencies of general users in the AI domain and has been validated and applied in populations such as students, teachers, and nurses [25–27]. The scale comprises a total of 12 items, all of which are rated on a 7‐point Likert‐type scale (1 = *strongly disagree*, 7 = *strongly agree*). Three items are reverse‐scored, and total scores range from 12 to 84, with higher scores reflecting greater AI literacy. In the present study, the overall Cronbach’s *α* coefficient for the scale was 0.884. The scale’s KMO value was 0.893, and Bartlett’s test of sphericity showed a significance level of < 0.001, indicating excellent internal consistency and structural validity for the adapted Chinese version, making it suitable for this study.

#### 2.5.4. Artificial Intelligence Self‐Efficacy Scale

The Artificial Intelligence Self‐Efficacy Scale [28] is designed to assess individuals’ perceived self‐efficacy regarding specific features of AI technology. This scale has been widely validated and applied in populations such as students and educators [29]. The scale comprises 22 items, which are divided into four dimensions: Assistance, Anthropomorphic Interaction, Comfort with AI, and Technological Skills. All items are rated on a 7‐point Likert‐type scale (1 = *strongly disagree*, 7 = *strongly agree*). Total scores range from 22 to 154, with higher scores reflecting stronger self‐efficacy in using AI technologies and products. In the present study, the overall Cronbach’s *α* coefficient for the scale was 0.979. The scale’s KMO value was 0.969, and Bartlett’s test of sphericity showed a significance level of < 0.001, indicating excellent internal consistency and structural validity for the adapted Chinese version, making it suitable for this study.

#### 2.5.5. Artificial Intelligence Anxiety Scale

The Artificial Intelligence Anxiety Scale [30] was used to assess nurses’ anxiety levels toward AI. The scale encompasses four dimensions: Learning Anxiety, Occupational Replacement Anxiety, Social‐Technological Blindness, and AI Configuration Anxiety. It consists of 21 items, all of which are rated on a 7‐point Likert scale (1 = *strongly disagree*, 7 = *strongly agree*), with total scores ranging from 21 to 147. Higher scores reflect more severe anxiety levels. In the present study, the Cronbach’s *α* coefficient for the overall scale was 0.974. The scale’s KMO value was 0.960, and Bartlett’s test of sphericity showed a significance level of < 0.001, indicating excellent internal consistency and structural validity for the adapted Chinese version, making it suitable for this study.

### 2.6. Data Analysis

Initial analyses examined the distribution and outliers of all variables. No missing data were present for any included variables, as the questionnaire was configured to prohibit submission with incomplete responses. Prior to LPA, four‐factor confirmatory factor analysis (CFA) was conducted to examine the convergent and discriminant validity of the four core constructs so as to verify that these latent variables were statistically distinguishable and free from serious construct overlap. Convergent validity was evaluated via composite reliability (CR) and average variance extracted (AVE), with thresholds of CR ≥ 0.70 and AVE ≥ 0.50 [31]. Discriminant validity was assessed using the HTMT_2_ criterion. HTMT_2_ values below 0.85 were considered to support adequate discriminant validity between paired constructs [32, 33]. LPA was performed to identify latent profiles based on nurses’ attitudes toward AI, AI literacy, AI self‐efficacy, and AI anxiety, and to determine the optimal number of classes according to model fit indices. Prior to LPA, the total scores of the four scales were respectively converted into standardized *Z*‐scores to eliminate the bias caused by inconsistent score ranges across measurement tools and ensure equal contribution of each indicator to profile classification. The model fit evaluation indices included the Akaike information criterion (AIC), Bayesian information criterion (BIC), adjusted BIC (aBIC), entropy, the Lo–Mendell–Rubin adjusted likelihood ratio test (LMRT), and the bootstrapped likelihood ratio test (BLRT). Model fit criteria were as follows: lower AIC, BIC, and aBIC values, together with higher entropy values, indicated better model fit. Statistically significant *p* values for the LMRT and BLRT suggested that the *k*‐class model was superior to the *k* − 1 class model [34, 35]. The best‐fitting model was selected based on the above criteria, alongside a heuristic minimum class size threshold of no less than 5% of the total sample to ensure statistical stability of subgroups [36, 37]. Notably, a fundamental assumption of LPA is that the probability distribution of responses to observed variables can be explained by a set of mutually exclusive latent classes, each characterized by a distinct response pattern across the observed variables.

Associations between work‐related factors or other covariates and latent clusters were examined using chi‐square tests. Subsequently, these factors were entered into a multivariate model using multinomial logistic regression analysis to explore distinct risk factor profiles within each cluster. CFA was conducted using the R‐lavaan package; LPA was performed using Mplus Version 8.3 (Muthén & Muthén, College Station, TX, USA), and all other statistical analyses were conducted using Stata Version 19 (StataCorp LP, College Station, TX, USA). The level of statistical significance was set at *p* < 0.05. This study was not registered in a public clinical trial registry.

## 3. Results

### 3.1. Sample Characteristics

A total of 1159 questionnaires were collected, of which 141 were excluded due to invalid responses (82 with abnormal completion times and 59 from nurses in rotation training or rehired after retirement), yielding 1018 valid responses (effective rate: 87.8%). Data were exported to Excel for cleaning and analysis.

The final sample of 1018 nurses was divided into Profile 1 (*n* = 680), Profile 2 (*n* = 212), and Profile 3 (*n* = 126). The sample was predominantly female (94.2%), Han ethnicity (92.4%), and married (71.0%), with the largest age group being 31–40 years (46.5%). Most held a bachelor’s degree (72.6%) and primary and below professional titles (42.6%). Over 90% engaged in clinical nursing, mainly in the pediatrics department (47.7%). Additionally, 56.1% worked as preceptors, and 44.2% had no AI training experience at all. Geographically, 82.9% were affiliated with western China, and the majority worked in Grade III hospitals (73.7%) and teaching hospitals (70.9%). Table 1 presents the full demographic and occupational characteristics of the participants.

**TABLE 1:** Comparison of characteristics among latent profiles in participating nurses *n* (%).

Variables	Total (*N* = 1018)	Profile 1 (*n* = 680)	Profile 2 (*n* = 212)	Profile 3 (*n* = 126)	*χ* ^2^	*p*
*Gender*						
Male	59 (5.8)	39 (5.7)	14 (6.6)	6 (4.8)	0.505	0.777
Female	959 (94.2)	641 (94.3)	198 (93.4)	120 (95.2)		

*Age group (years)*						
≤ 30	395 (38.8)	266 (39.1)	85 (40.1)	44 (34.9)	3.535	0.739
31–40	473 (46.5)	320 (47.1)	91 (42.9)	62 (49.2)		
41–50	132 (13.0)	81 (11.9)	33 (15.6)	18 (14.3)		
≥ 51	18 (1.8)	13 (1.9)	3 (1.4)	2 (1.6)		

*Ethnicity*						
Han	941 (92.4)	631 (92.8)	191 (90.1)	119 (94.4)	2.514	0.284
Other	77 (7.6)	49 (7.2)	21 (9.9)	7 (5.6)		

*Marital status*						
Unmarried	269 (26.4)	177 (26.0)	65 (30.7)	27 (21.4)	4.931	0.294
Married	723 (71.0)	488 (71.8)	141 (66.5)	94 (74.6)		
Divorced	26 (2.6)	15 (2.2)	6 (2.8)	5 (4.0)		

*Education level*						
Associate degree or below	239 (23.5)	164 (24.1)	54 (25.5)	21 (16.7)	**20.174**	**< 0.001**
Bachelor’s degree or undergraduate	739 (72.6)	501 (73.7)	144 (67.9)	94 (74.6)		
Master’s degree or above	40 (3.9)	15 (2.2)	14 (6.6)	11 (8.7)		

*Professional title*						
None	106 (10.4)	73 (10.8)	27 (12.7)	6 (4.8)	**16.070**	**0.014**
Primary and below	434 (42.6)	287 (42.1)	85 (40.1)	62 (49.2)		
Intermediate	411 (40.4)	285 (41.9)	77 (36.3)	49 (38.9)		
Associate senior and above	67 (6.6)	35 (5.2)	23 (10.9)	9 (7.1)		

*Years of work experience (years)*						
≤ 5	246 (24.2)	169 (24.9)	59 (27.8)	18 (14.3)	**18.568**	**0.005**
6–10	273 (26.8)	179 (26.3)	45 (21.2)	49 (38.9)		
11–15	277 (27.2)	189 (27.7)	62 (29.3)	27 (21.4)		
≥ 16	222 (21.8)	144 (21.1)	46 (21.7)	32 (25.4)		

*Department*						
Pediatrics	486 (47.7)	309 (45.4)	103 (48.6)	74 (58.7)	**14.956**	**0.021**
Obstetrics and gynecology	270 (26.5)	184 (27.1)	53 (25.0)	33 (26.2)		
Non‐inpatient department	130 (12.8)	99 (14.6)	21 (9.9)	10 (7.9)		
Adult general	132 (13.0)	88 (12.9)	35 (16.5)	9 (7.2)		

*Job content*						
Administration/teaching/research/other	76 (7.5)	47 (6.9)	15 (7.1)	14 (11.1)	2.773	0.250
Clinical nursing	942 (92.5)	633 (93.1)	197 (92.9)	112 (88.9)		

*Preceptor*						
Yes	571 (56.1)	364 (53.5)	124 (58.5)	83 (65.9)	**7.201**	**0.027**
No	447 (43.9)	316 (46.5)	88 (41.5)	43 (34.1)		

*AI training experience*						
No training received whatsoever	450 (44.2)	331 (48.7)	70 (33.0)	49 (38.9)	24.905	< 0.001
No professional training but received related thematic training or self‐study	423 (41.6)	273 (40.2)	97 (45.8)	53 (42.1)		
Received professional training	145 (14.2)	76 (11.1)	45 (21.2)	24 (19.0)		

*Region of affiliation*						
Eastern China	112 (11.0)	58 (8.5)	30 (14.2)	24 (19.1)	**19.267**	**0.001**
Central China	62 (6.1)	47 (6.9)	6 (2.8)	9 (7.1)		
Western China	844 (82.9)	575 (84.6)	176 (83.0)	93 (73.8)		

*Hospital type*						
Specialty hospital	516 (50.7)	340 (50.0)	108 (50.9)	58 (46.0)	0.677	0.713
General hospital	502 (49.3)	340 (50.0)	104 (49.1)	68 (54.0)		

*Hospital level*						
Grade III	750 (73.7)	481 (70.7)	159 (75.0)	110 (87.3)	**15.284**	**< 0.001**
Grade II or below	268 (26.3)	199 (29.3)	53 (25.0)	16 (12.7)		

*Teaching hospital*						
Yes	722 (70.9)	469 (69.0)	148 (69.8)	105 (83.3)	**10.794**	**0.005**
No	296 (29.1)	211 (31.0)	64 (30.2)	21 (16.7)		

*Note:* Bold values indicate statistically significant differences (*p* < 0.05).

### 3.2. Measurement Model Assessment

Prior to LPA, four‐factor CFA was conducted to verify the convergent and discriminant validity of the four constructs. The results showed that CR of the four constructs ranged from 0.925 to 0.980, and AVE ranged from 0.611 to 0.692, both satisfying the recommended thresholds of CR ≥ 0.70 and AVE ≥ 0.50. The four‐factor measurement model yielded scaled *χ* ^2^ = 9307.428, *df* = 2670, scaled *χ* ^2^ */df* = 3.486, robust CFI = 0.885, robust TLI = 0.881, robust RMSEA = 0.065 (90% CI = 0.064–0.066), and SRMR = 0.140. To further examine discriminant validity, a competing three‐factor model was tested, in which AI literacy and AI self‐efficacy were loaded onto a single latent factor. The three‐factor alternative model produced scaled *χ* ^2^ = 9651.697, *df* = 2673, scaled *χ* ^2^ */df* = 3.611, robust CFI = 0.878, robust TLI = 0.874, robust RMSEA = 0.067 (90% CI = 0.066–0.068), SRMR = 0.139. Nested‐model comparison showed that the four‐factor model fitted the data significantly better than the three‐factor model (Δ*χ* ^2^ = 44.857, Δ*df* = 3, *p* < 0.001).

HTMT_2_ ratios were calculated to evaluate discriminant validity, with a threshold value of 0.85 applied. The pairwise HTMT_2_ values were as follows: AI attitude–AI literacy = 0.694; AI attitude–AI self‐efficacy = 0.748; AI attitude–AI anxiety = 0.156; AI literacy–AI self‐efficacy = 0.793; AI literacy–AI anxiety = 0.234; AI self‐efficacy–AI anxiety = 0.145. All pairwise HTMT_2_ values were below 0.85, supporting acceptable discriminant validity across constructs. Collectively, these results indicated no serious construct overlap among the four latent variables and supported the reliability of subsequent LPA.

### 3.3. Latent Profiles of AI Attitude, Literacy, Self‐Efficacy, and Anxiety

In this study, five latent profile models were fitted, and the fitting indices of each model are shown in Table 2. Although AIC, BIC, and aBIC decreased steadily as more latent profiles were specified, the reduction in BIC values gradually slowed after the three‐profile model. Specifically, BIC declined by 192.73 between the 3‐ and 4‐profile models, 143.64 between the 4‐ and 5‐profile models, and 86.59 between the 5‐ and 6‐profile models, which suggests marginal improvements in model fit become limited with additional subgroups. Entropy values for all candidate models exceeded 0.80, and the 3‐profile model yielded the highest entropy (0.893), indicating better separation across subgroups. The LMR likelihood ratio test was statistically significant for the 2‐, 3‐, and 5‐profile models (*p* < 0.05). The 2‐profile solution was set aside due to its substantially higher BIC (10736.22) and lower entropy (0.839) relative to the 3‐profile model. The LMR test was nonsignificant for both the 4‐profile (*p* = 0.103) and 6‐profile models (*p* = 0.234). The 4‐profile model was therefore not retained, given its nonsignificant LMR and relatively lower entropy (0.821) than the 3‐profile model. The 6‐profile model was also considered less suitable, owing to its nonsignificant LMR, two subgroups falling below the 5% stability threshold (2.5% and 2.4%), and only a marginal reduction in BIC compared with the 5‐profile model (86.59). Although the 5‐profile model returned a significant LMR result, it was not retained due to a small subgroup (3.0% of the sample), highly congruent score patterns across the four indicators, and only a moderate entropy value of 0.881. Considering marginal fit gains, entropy‐derived classification precision, LMR test statistics, subgroup heterogeneity, and the minimum class size criterion, the 3‐profile model was identified as the optimal solution. Mean score distributions of the three latent profiles across the four study indicators are presented in Figures 1 and 2.

**TABLE 2:** Fit indices of latent profile analysis and distribution rate.

Cluster	AIC	BIC	SaBIC	Entropy	LMR	BLRT	Latent profile distribution rate (%)					
							1	2	3	4	5	6
2‐profile	10672.16	10736.22	10694.93	0.839	< 0.001	< 0.001	65.0	35.0				
3‐profile	10172.23	10260.92	10203.76	0.893	< 0.001	< 0.001	66.9	20.8	12.3			
4‐profile	9954.86	10068.19	9995.14	0.821	0.103	< 0.001	52.8	7.7	27.0	12.5		
5‐profile	9786.57	9924.55	9835.62	0.881	0.001	< 0.001	57.7	10.7	6.3	22.3	3.0	
6‐profile	9675.35	9837.96	9733.15	0.888	0.234	< 0.001	56.2	11.0	5.4	22.5	2.5	2.4

**FIGURE 1:**
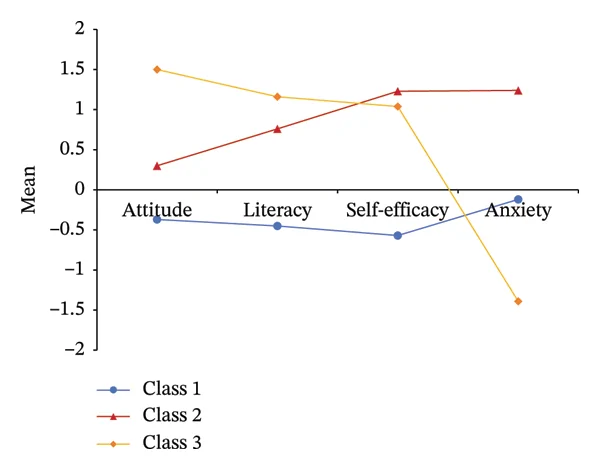
Mean *Z*‐scores of AI attitude, literacy, self‐efficacy, and anxiety scores by cluster. Profile 1: low AI perceptions and readiness profile; Profile 2: ambivalent AI perceptions and readiness profile; Profile 3: high AI perceptions and readiness profile.

**FIGURE 2:**
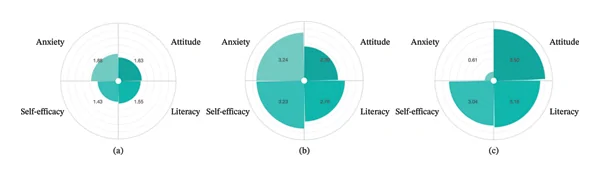
Mean *Z*‐scores of AI attitude, literacy, self‐efficacy, and anxiety scores by cluster; all *Z* values were added by 2 to eliminate negative values for polar visualization. (a) Profile 1. (b) Profile 2. (c) Profile 3.

Profile 1 had the lowest scores in AI attitude, AI literacy, AI self‐efficacy, and moderate AI anxiety. We named this group the “Low AI Perceptions and Readiness Profile,” which reflects nurses’ relatively low overall psychological recognition of AI technology. This group accounted for 66.80% of the sample (*n* = 680).

Profile 2 showed a moderate score in AI attitude and a moderate score in AI literacy. Meanwhile, this class had the highest score in AI self‐efficacy among the three classes, yet also scored highest in AI anxiety. We named this group the “Ambivalent AI Perceptions and Readiness Profile.” This subgroup was characterized by a moderate AI cognitive foundation and a strong self‐perceived ability to operate AI tools, accompanied by the most prominent AI‐related psychological anxiety, showing typical contradictory psychological characteristics. It comprised 20.83% of the sample (*n* = 212).

Profile 3 showed the highest scores in AI attitude and AI literacy, with a high score in AI self‐efficacy and the lowest score in AI anxiety among the three classes. We named this group the “High AI Perceptions and Readiness Profile.” It represented nurses with a positive and receptive attitude toward AI, high AI literacy and self‐efficacy, and minimal psychological anxiety about AI perceptions and readiness. This group represented 12.37% of the sample (*n* = 126).

### 3.4. Distinct Profiles of Female Nurses in the Three Clusters

The relationships between AI use and nurses’ demographic characteristics, work‐related factors, and AI training experience are shown in Table 1.

Nurses in Profile 1 (Low AI Perceptions and Readiness Profile) had the lowest proportion of individuals with a master’s degree or higher (2.2%), the lowest proportion of those serving as preceptors (53.5%), and the lowest proportion affiliated with eastern China (8.5%), as well as the lowest proportions of employment in Grade III hospitals (70.7%) and teaching hospitals (69.0%) compared with the other two classifications (Table 1).

Nurses in Profile 2 (Ambivalent AI Perceptions and Readiness Profile) exhibited a moderate proportion of master’s degree holders (6.6%), the highest proportion of individuals with an associate degree or below (25.5%), and the highest proportion with less than 5 years of work experience (27.8%) compared with the other two classifications (Table 1). Compared to those in Profile 1, nurses in Profile 2 were more likely to have AI‐related training, work in Grade III hospitals, and be employed in nonteaching hospitals (Figure 3).

**FIGURE 3:**
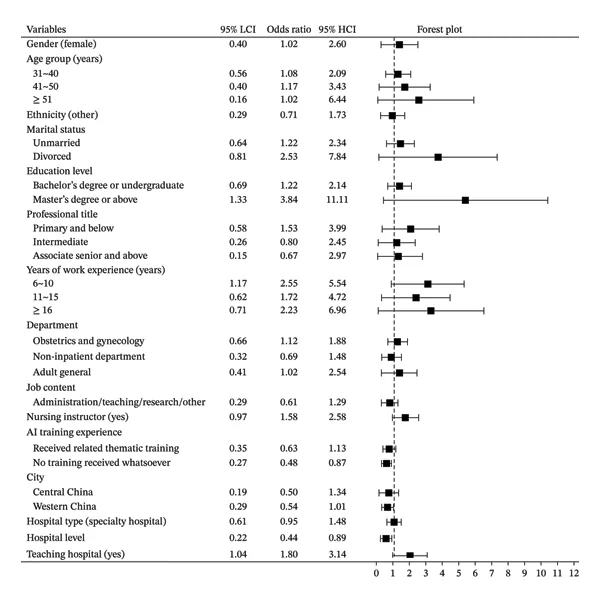
Multinomial logistic regression analysis showing the association of influencing factors for Profile 2 compared to Profile 1.

Nurses in Profile 3 (High AI Perceptions and Readiness Profile) had the highest proportion of master’s degree holders (8.7%), the highest proportion with junior professional titles (49.2%), the highest proportion with 6 to 10 years of work experience (38.9%), the highest proportion working in pediatrics (58.7%), the highest proportion serving as preceptors (65.9%), and the highest proportion affiliated with eastern China (19.1%), as well as the highest proportions of employment in Grade III hospitals (87.3%) and teaching hospitals (83.3%) compared with the other two classifications (Table 1). Compared to those in Profile 1, nurses in Profile 3 were more likely to have received AI‐related training, work in eastern China, and be employed in Grade III hospitals (Figure 4).

**FIGURE 4:**
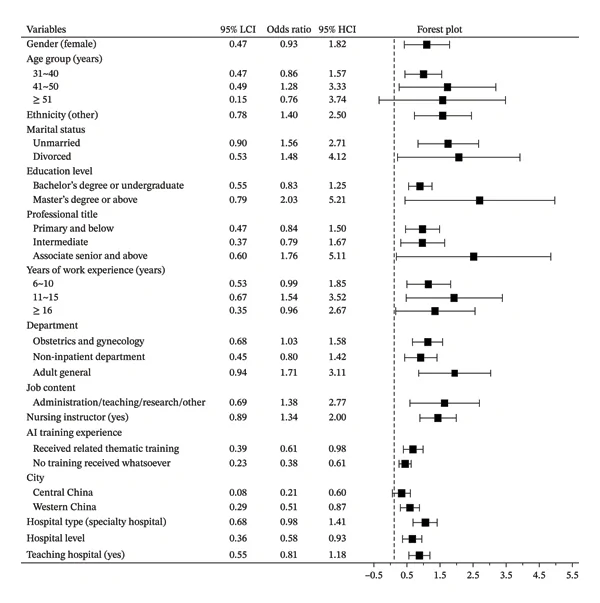
Multinomial logistic regression analysis showing the association of influencing factors for Profile 3 compared to Profile 1.

## 4. Discussion

This study adopted LPA to categorize 1018 clinical nurses into three heterogeneous subgroups based on four core dimensions: attitudes toward AI, AI literacy, AI self‐efficacy, and AI‐related anxiety. The three distinct profiles were labeled the Low AI Perceptions and Readiness Profile, the Ambivalent AI Perceptions and Readiness Profile, and the High AI Perceptions and Readiness Profile, accounting for 66.80%, 20.83%, and 12.37% of the total sample, respectively. This classification clearly reveals the heterogeneity in clinical nurses’ AI perceptions and readiness, with significant intergroup differences detected in core psychological traits, demographic characteristics, and occupational attributes.

### 4.1. Characteristics of Each AI Perceptions and Readiness Subgroup and Associations With Demographic and Occupational Factors

#### 4.1.1. The Low AI Perceptions and Readiness Profile: Features of the Dominant Low Adaptation Group

The Low AI Perceptions and Readiness Profile represented the largest subgroup in this study, encompassing more than 60% of participants. Nurses in this profile scored the lowest on three core indicators, including attitudes toward AI, AI literacy, and AI use self‐efficacy, and reported only moderate AI‐related anxiety, manifesting a typical pattern of resistance to AI technology, inadequate competence, and low confidence. Demographically and occupationally, merely 2.2% held a master’s degree or higher, a proportion markedly lower than the other two subgroups. This profile also had the lowest percentages of nurse educators, nurses practicing in eastern China, and those employed in Grade III and teaching hospitals. Notably, 48.7% had no prior AI‐related training experience, making this subgroup the most underserved in AI training coverage among the three identified profiles.

Several interrelated factors explain this characteristic pattern. Most nurses in this profile worked in primary‐care or nonteaching hospitals with limited medical resources, where AI technology penetration and access to intelligent nursing tools are scarce. These findings are consistent with previous studies [38, 39], which identified significant contextual challenges to implementing AI in healthcare among low‐resource environments.

Long‐term lack of systematic, specialized AI training had led to insufficient AI knowledge reserves and poor practical skills, fostering a fearful mindset of being unable to operate and afraid to apply AI, alongside a conservative, resistant attitude toward its adoption. Furthermore, the majority held junior or lower professional titles and had relatively short clinical tenure, burdened by heavy routine basic nursing workloads that left little time for proactive AI learning. This further intensified their conservative stance on AI perceptions and readiness, positioning this subgroup as a key barrier to the widespread integration of AI technology in nursing practice.

#### 4.1.2. The Ambivalent AI Perceptions and Readiness Profile: Features of the Anxious Intermediate Transitional Group

The Ambivalent AI Perceptions and Readiness Profile accounted for 20.83% of the sample, serving as the intermediate subgroup. Nurses in this profile displayed moderate AI attitudes and AI literacy, the highest AI use self‐efficacy among all groups, and concurrently the most severe AI‐related anxiety, presenting a contradictory state of “having basic competence and willingness to try, yet deeply apprehensive.” Demographically and occupationally, this subgroup had the highest proportion of nurses with a junior college degree or lower. In addition, 27.8% were young nurses with 5 years or fewer of clinical experience, representing the highest proportion among the three subgroups. The percentage of nurses with AI‐related training and those working in Grade III hospitals was significantly higher than that of the Low AI Perceptions and Readiness Profile, whereas the proportion employed in nonteaching hospitals was relatively elevated.

Most nurses in this subgroup were frontline young nurses. Although they had completed basic AI training or self‐directed learning, gaining preliminary AI application skills and strong self‐efficacy with some willingness to adopt AI, they were limited by lower educational attainment and an unsystematic AI knowledge base. Working on the clinical frontline, they were acutely aware of operational challenges, data security risks, and potential career replacement threats linked to AI use, triggering prominent anxiety. These results are consistent with previous studies [40, 41]. As a result, they held a cautious, wait‐and‐see attitude toward AI. They neither fully reject new technology nor actively engage in in‐depth AI application, staying in a passive, ambivalent psychological state. This group presents moderate levels of AI perceptions and readiness among nurses, and the anxiety observed within this subgroup highlights the necessity of carrying out targeted AI training interventions for this population.

#### 4.1.3. The High AI Perceptions and Readiness Profile: Features of the High Adaptation Benchmark Group

The High AI Perceptions and Readiness Profile was the smallest subgroup, representing only 12.37% of the sample, yet demonstrated the highest adaptability to AI application. Nurses in this profile achieved the highest scores in AI attitudes, AI literacy, and AI use self‐efficacy, with the lowest AI‐related anxiety, reflecting a proactive, positive stance toward AI adoption and implementation. Regarding key characteristics, this subgroup recorded the highest percentages of favorable occupational attributes: 8.7% held a master’s degree or higher, 49.2% were nurse practitioners, 38.9% had 6 to 10 years of clinical experience, 58.7% practiced in pediatric departments, 65.9% were nurse educators, 19.1% worked in eastern China, 87.3% were in Grade III hospitals, and 83.3% were in teaching hospitals. The share of nurses with specialized AI training was also notably higher than the other two subgroups.

Nurses in this profile were mostly core clinical backbones with high education levels and moderate clinical experience, employed in Grade III teaching hospitals with concentrated high‐quality medical resources, and some also held nursing education roles. These characteristics are consistent with previous research [39, 42]. They possessed exceptional professional literacy and learning capacity, worked in settings with diverse AI application scenarios, and had access to systematic, specialized AI training. Equipped with solid AI literacy and practical skills, they had a clear understanding of AI’s advantages and clinical value, thus exhibiting high confidence in AI use with minimal anxiety, and actively integrated intelligent tools into routine clinical nursing. This subgroup serves as a core benchmark and pivotal force for promoting AI technology in nursing. Meanwhile, differences in departmental composition across latent profiles may reflect varied needs and acceptance tendencies regarding AI technologies among nursing staff in different clinical contexts.

### 4.2. Limitations

This study has several key limitations. First, questionnaires were distributed nationwide with assistance from the Chinese Nursing Association, yet 82.9% of participants were recruited from western China. Constrained by our institutional background, the questionnaire was distributed primarily among maternal and child nursing staff, resulting in a skewed sample composition and restricting the external generalizability of our results. Second, the cross‐sectional design precludes causal inference and fails to capture longitudinal transitions of latent profiles. Third, only limited individual and institutional covariates were incorporated into the analysis, while multilevel contextual variables at the organizational level were not included. Additionally, the tiered questionnaire administration may induce correlated observations within the same medical institution, which implies potential clustering effects that could bias statistical outcomes. These two aspects collectively impair the explanatory robustness of the statistical model. Fourth, the AI self‐efficacy and AI anxiety scales exhibited extremely high Cronbach’s *α* values, implying strong inter‐item homogeneity and potential item redundancy among scale items. Fifth, this study did not carry out targeted intervention programs, so the practical effect of classified and hierarchical training strategies cannot be verified. Finally, the measurement model yielded marginal CFA fit indices, which may be attributable to the relatively large number of items included in the full scale. Nevertheless, supplementary nested‐model comparison and established HTMT_2_ criteria consistently supported adequate discriminant validity across the four constructs.

To address these limitations, future research should (1) employ probability sampling strategies to ensure more representative and diverse samples across different regions and hospital tiers; (2) adopt longitudinal or quasi‐experimental designs to examine causal relationships and track competency development over time; (3) incorporate multilevel organizational contextual and institutional characteristic variables to systematically identify facilitators and barriers of AI adoption and implement corresponding statistical adjustments; (4) appropriately screen items and develop simplified scales to improve questionnaire efficiency while maintaining measurement quality; and (5) conduct rigorously designed intervention studies to evaluate the effectiveness of stratified training programs in real‐world educational and clinical settings.

## 5. Implications for Nursing Management

This study identifies three distinct AI perceptions and readiness profiles among nurses, offering empirical reference for the design of targeted educational interventions in nursing training programs.

For the largest group, the Low AI Perceptions and Readiness Profile, the data indicate limited baseline knowledge. This observation raises the hypothesis that foundational literacy training, delivered through clinically anchored stepwise modules, could reduce cognitive overload. The potential benefit of protected hands‐on practice in resource‐limited settings is an empirical question that falls beyond the reach of the current design. Similarly, the optimal training dosage and format cannot be determined from the present data and thus warrant systematic investigation in future experimental studies.

For the Ambivalent AI Perceptions and Readiness Profile, the findings suggest a transitional state characterized by high anxiety alongside high self‐efficacy. This pattern points to the hypothesis that interventions targeting uncertainty reduction and efficacy reinforcement, such as real‐world case scenarios and interactive workshops, may be particularly relevant. Whether such pedagogical approaches effectively reduce AI anxiety remains to be established through longitudinal or quasi‐experimental designs.

For the High AI Perceptions and Readiness Profile, this group’s positive orientation suggests a peer‐mentoring or early‐adopter role. Co‐facilitating workshops may reinforce their expertise and provide peer support to less confident colleagues. Advanced training tracks could develop their AI leadership potential, though the effectiveness of such models requires empirical validation.

Overall, this stratified framework provides an empirically grounded foundation for designing needs‐based training strategies tailored to distinct nurse profiles, offering nurse educators and managers a practical tool for curriculum planning and resource allocation.

## 6. Conclusions

This study identified three latent profiles of AI perceptions and readiness among clinical nurses using LPA: the Low AI Perceptions and Readiness Profile, the Ambivalent AI Perceptions and Readiness Profile, and the High AI Perceptions and Readiness Profile. Significant demographic and occupational differences were observed across the three subgroups.

The Low AI Perceptions and Readiness Profile accounted for the largest proportion of participants, and members of this group reported limited access to relevant training and educational resources. The Ambivalent AI Perceptions and Readiness Profile was characterized by relatively high anxiety and hesitant attitudes toward clinical AI application. Participants in the High AI Perceptions and Readiness Profile showed favorable competence and positive recognition of AI technologies within clinical workflows.

The findings offer empirical reference for developing profile‐based differentiated training to meet nurses’ diverse learning needs and support the uptake of AI in nursing education and clinical practice. Given the cross‐sectional design and limited sample representativeness, further studies are needed to verify the generalizability of these results. Future longitudinal and intervention research may explore the value of tailored educational strategies for nursing digital transformation.

## Author Contributions

Bei Yang: conception and design of the study, statistical analysis, drafting the article, and performed the manuscript review.

Jiacheng Hu: acquisition and interpretation of data, and performed the manuscript review.

Qin Zeng: conception and design of the study, acquisition and interpretation of data, and final approval of the version to be published.

## Funding

This study was supported by the 11th Phase Higher Education Teaching Reform Project of Sichuan University (Grant No. SCU1199).

## Ethics Statement

All procedures performed in studies involving human participants were in accordance with the ethical standards of the researchers’ affiliated institution [Ethics approval number: Medical Research 2025 Ethics Approval No. (050)].

## Conflicts of Interest

The authors declare no conflicts of interest.

## Supporting Information

Additional supporting information can be found online in the Supporting Information section.

## Supporting information


**Supporting Information** Strobe checklist.

## Data Availability

Data from this study will be shared with qualified investigators upon reasonable request for scientific purposes.
